# Mechanically Exfoliated Multilayer Graphene-Supported Ni-MOF Parallelogram Nanosheets for Enhanced Supercapacitor Performance

**DOI:** 10.3390/nano15090643

**Published:** 2025-04-23

**Authors:** Zhiheng Li, Junming Xu, Xinqi Ding, Haoran Zhu, Jianfeng Wu

**Affiliations:** 1 College of Electronic Information, Hangzhou Dianzi University, Hangzhou 310018, China; 231040046@hdu.edu.cn (Z.L.); 232040319@hdu.edu.cn (X.D.); 232040345@hdu.edu.cn (H.Z.); 2 College of Information Science & Technology, Zhejiang Shuren University, Hangzhou 310015, China

**Keywords:** supercapacitor, metal-organic frameworks, Ni-MOF, multilayer graphene

## Abstract

Metal–organic frameworks (MOFs) are regarded as advanced supercapacitor materials owing to their high surface area, redox-active sites, and porosity. However, their insufficient charge carrier mobility remains a critical limitation for practical application. Integrating MOFs with conductive carbon substrates is an effective strategy to break through this limitation. However, conventional carbon materials often require complex preparation methods and pre-activation steps for use in MOF composites. Herein, multilayer graphene (MLG) mechanically exfoliated from expandable graphite is employed as a substrate, and a van der Waals force-assisted chemical deposition method is developed to directly anchor Ni-MOF onto its surface without requiring pre-activation treatment. To optimize the composite, Ni-MOFs with various mass loadings are synthesized on MLG surface. The morphological characteristics and energy storage performance of these composites are thoroughly characterized. Ni-MOF/MLG-0.30 (with a 70.8% Ni-MOF loading on MLG) features a porous stacking structure of well-crystalline Ni-MOF parallelogram nanosheets on MLG, exhibiting optimal electrochemical performance. The composite achieves 1071.4 F·g^−1^ at 1 A·g^−1^, and a capacitance retention of 64.9% at the elevated current density of 10 A·g^−1^. Meanwhile, the composite maintains 63.2% of its initial capacitance after 5000 charge/discharge cycles at 4 A·g^−1^. A hybrid supercapacitor is fabricated using Ni-MOF/MLG-0.30 cathode and activated carbon anode, delivering 27.9 Wh·kg^−1^ energy density at 102.5 W·kg^−1^ power output.

## 1. Introduction

Supercapacitors have gained widespread attention as next-generation energy storage solutions due to their exceptional power density, long cycle life, and rapid charge/discharge capabilities. These properties enable their use in diverse fields such as electric vehicles, renewable energy systems, and consumer electronics [1,2]. However, supercapacitors face a key obstacle in insufficient energy density for applications requiring greater storage capacity [3]. The energy storage behavior of supercapacitors enables their division into three classes: electric double-layer capacitors (EDLCs), pseudocapacitors, and battery-type capacitors. The energy storage mechanism in battery-type capacitors relies on redox reactions occurring throughout the bulk electrode material, enabling significantly higher energy density compared to EDLCs (which rely on physical adsorption of charges at the solid–liquid interface) and pseudocapacitors (where redox reactions are limited to surface and near-surface atomic layers without bulk ion diffusion) [4]. A variety of metal compounds, including metal oxides [5], hydroxides [6], sulfates [7], phosphates [8], and metal–organic frameworks (MOFs) [9,10,11,12,13,14], have been investigated as potential electrode candidates for battery-type capacitors. Metal–organic frameworks (MOFs) represent a distinctive category of porous crystalline materials formed through coordination bonding between metallic nodes (ions or clusters) and multidentate organic linkers, generating well-defined two- or three-dimensional (3D) network structures [15]. Among these, nickel-based MOFs (Ni-MOFs) show great potential for supercapacitors due to their highly reversible redox reactions and their tunable porous frameworks, which facilitate efficient ion transport and charge storage.

The primary limitation hindering the application of Ni-MOFs in supercapacitors is their inherently low electrical conductivity. To address this challenge, researchers have proposed several strategies, such as synthesizing bimetallic MOFs or converting MOFs into porous carbon or metal compounds, which can enhance their electrochemical performance [16]. However, these methods often incur high costs and compromise the intrinsic functionality of the MOFs. An alternative strategy involves integrating MOFs with conductive substrates, among which carbon-based materials are the most widely utilized. Carbon nanotubes [17,18] and graphene [19,20,21,22,23,24] stand out because of their exceptional surface-to-volume ratios and electrical conductivity. However, the chemical inertness of pristine carbon materials requires pre-activation steps, such as surface functionalization or defect engineering, to ensure strong interfacial bonding between the carbon substrate and MOFs.

Yazdani et al. [25] functionalized multi-walled carbon nanotubes (MWCNTs) by dispersing them in nitric acid and refluxing the mixture at 110 °C for 6 h. Subsequently, Ni-MOF particles were grown on the surface of the functionalized MWCNTs via coordinate bonding between Ni^2+^ ions and carboxyl groups (-COOH) introduced on the MWCNTs, using a hydrothermal synthesis method at 150 °C for 18 h. An as-prepared Ni-MOF/MWCNT composite achieved 900 F·g^−1^ at 0.5 A·g^−1^, maintaining 82% of its initial capacity through 1000 cycles. Jin et al. [26] employed graphene oxide (GO) as the carbon source, synthesized via an improved Hummers method. The synthesis involved treating graphite powder with a H_2_SO_4_/H_3_PO_4_ mixture in an oil bath, followed by the treatment of KMnO_4_ at 50 °C for 6 h, and the dropwise addition of H_2_O_2_. Numerous defects and oxygen-containing functional groups were introduced into graphene. Then, Zn/Ni-MOF/rGO composite was subsequently prepared using a solvothermal method at 120 °C for 12 h. The optimized composite demonstrated 1644 F·g^−1^ at 1 A·g^−1^ and a capacitance retention of 85.1% after 3000 cycles. Xiao et al. [27] prepared high-quality graphene via liquid-phase exfoliation of graphite in a solvent. Numerous oxygen-containing surface functionalities were introduced onto the graphene by grafting glucose derivatives onto the liquid-exfoliated graphene (LEG) surface through a simple solvothermal process. Subsequently, Ni-MOF was synthesized on the graphene via solvothermal treatment at 100 °C for 8 h. The Ni^2+^ precursor was anchored onto the surface of LEG via strong electrostatic interactions. Subsequent coordination with PTA (terephthalic acid) ligands to the Ni^2+^ ions facilitated the in situ transformation into Ni-MOF on the graphene substrate. Electrochemical measurements revealed that the prepared composite delivered 987.6 F·g^−1^ at 0.5 A·g^−1^, coupled with high cycling stability evidenced by 85.6% capacitance preservation over 3000 cycles.

The introduction of functionalization and defects facilitates the anchoring of MOFs on carbon materials. However, these modifications significantly degrade the electrical conductivity of carbon substrates and complicate the composite preparation process, thereby increasing overall costs. In this work, multilayer graphene (MLG), prepared via a liquid exfoliation method, serves as a carbon substrate for the direct deposition of Ni-MOF without pre-activation. The simplicity of the MLG synthesis and the elimination of pre-activation steps reduce the production cost of the Ni-MOF/MLG composites. A chemical deposition method was developed in this synthesis to replace conventional hydrothermal approaches. The anchoring mechanism of Ni-MOF relies on van der Waals interactions between the Ni^2+^ complex and the MLG surface, rather than electrostatic forces. The resulting Ni-MOF parallelogram nanosheets are uniformly dispersed on the MLG surface. The MLG substrate not only mitigates Ni-MOF aggregation but also shortens ion/electron transport pathways and enhances overall electrical conductivity. Electrochemical characterization reveals that the synthesized Ni-MOF/MLG-0.30 composite achieves 1071.4 F·g^−1^ at 1 A·g^−1^, while maintaining 63.2% of its initial capacitance after 5000 cycles, demonstrating much higher electrochemical performance than MLG-free Ni-MOF.

## 2. Experimental Section

### 2.1. Materials

Nickel chloride hexahydrate (NiCl_2_·6H_2_O) was purchased from Shanghai Macklin Biochemical Technology Co., Ltd. (Shanghai, China). Expanded graphite was purchased from Qingdao Tianyuanda Graphite Co., Ltd. (Qingdao, China). Ammonia (NH_3_·H_2_O) was purchased from Hangzhou Gaojing Fine Chemical Co., Ltd. (Hangzhou, China) N,N-dimethylformamide (DMF) and terephthalic acid (PTA) were purchased from Sinopharm Group (Shanghai, China). All reagents were used without further purification.

### 2.2. Preparation of Ni-MOF/MLG

**Figure 1:**
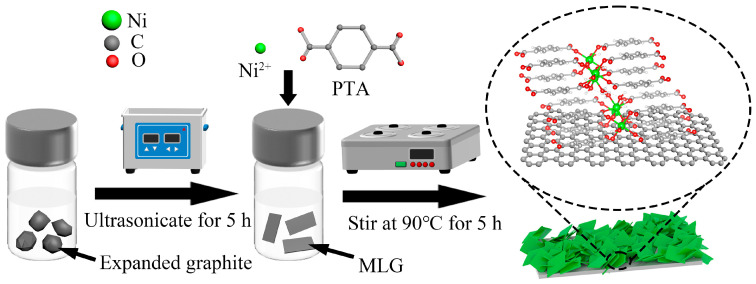
Schematic synthesis process of Ni-MOF parallelogram nanosheets on MLG.

Figure 1 illustrates the schematic synthesis process of Ni-MOF nanosheets on MLG. Expanded graphite features increased interlayer spacing (from ~0.34 nm to the micrometer scale) while maintaining its graphene-like layered units. Thus, the expanded architecture enables highly efficient exfoliation into MLG nanosheets, making expanded graphite an ideal precursor for MLG production. The detailed procedure is outlined as follows. First, 20 mg of expanded graphite was dispersed in a mixed solvent of 2 mL of deionized water and 8 mL of DMF. Ultrasonic treatment was applied to the mixture at 200 W power for 5 h to achieve graphite exfoliation, yielding a flat-surfaced MLG with minimal structural defects. Subsequently, equimolar amounts of NiCl_2_·6H_2_O and PTA were added to the MLG suspension. After 15 min of magnetic stirring, 200 mL of ammonium hydroxide (NH_3_·H_2_O) was introduced, followed by another 15 min of stirring. The homogeneous solution was then transferred to a 90 °C water bath and stirred magnetically for 5 h. Then, the vial was extracted from the bath and cooled to ambient temperature. The product was centrifugally washed repeatedly with anhydrous ethanol and deionized water. Finally, the dried product (80 °C oven) was collected and labeled as Ni-MOF/MLG-x, where x denotes the molar quantity (0.25, 0.30, 0.35, 0.40, or 0.45 mmol) of NiCl_2_·6H_2_O and PTA used. Gravimetric analysis revealed the Ni-MOF loading percentages on MLG across the series: Ni-MOF/MLG-0.25, -0.30, -0.35, -0.40, and -0.45 exhibited mass ratios of 63.7%, 70.8%, 72.3%, 77.2%, and 78.3%, respectively. For comparison, pure Ni-MOF-0.30 was synthesized under identical conditions to Ni-MOF/MLG-0.30 but without MLG.

### 2.3. Characterization

Structural characterization of the synthesized products was performed by X-ray diffraction (XRD, Shimadzu XRD-6000, Kyoto, Japan). Morphological evaluation was conducted through field-emission scanning electron microscopy (FESEM, Hitachi S-4800, Chiyoda City, Japan) and high-resolution transmission electron microscopy (HRTEM, JEOL-2100F, Tokyo, Japan). Chemical composition analysis included Fourier-transform infrared spectroscopy (FTIR, Nicolet iS10, Madison, WI, USA), and X-ray photoelectron spectroscopy (XPS, Thermo ESCALab 250Xi, Waltham, MA, USA) for surface chemistry analysis. Textural properties were assessed through nitrogen adsorption–desorption measurements, with specific surface area calculated using the BET method, and pore size distribution determined from adsorption isotherms via the BJH model.

### 2.4. Electrochemical Measurements

The working electrode was prepared by mixing the active material, conductive carbon, and polyvinylidene fluoride (PVDF) in a mass ratio of 7.5:1.5:1 using N-methyl-2-pyrrolidone (NMP) as the solvent. After stirring for 3 h to ensure homogeneity, the resulting slurry was uniformly coated onto pre-cleaned nickel foam substrates (1.0 × 1.0 cm^2^). The coated Ni foam was vacuum-dried at 80 °C for 24 h and subsequently pressed at 10 MPa for 1 min to enhance electrode integrity.

The electrochemical performance was assessed using a standard three-electrode configuration with 4.0 M KOH electrolyte, employing a CHI760E workstation. Cyclic voltammetry (CV) measurements spanned 5 to 50 mV·s^−1^ scan rates (0–0.6 V vs. Ag/AgCl), while galvanostatic charge–discharge (GCD) tests covered current densities from 1 to 10 A·g^−1^. Electrochemical impedance spectroscopy (EIS) analysis was carried out at 5 mV amplitude across 0.01 Hz to 10 kHz. Specific capacitance (Cs) values were derived from GCD data according to Equation (1):

(1) $$\begin{array}{c} C_{s}=\frac{I\times \Delta t}{m\times \Delta V} \end{array}$$

Here, *C_s_* is specific capacitance (F·g^−1^), *I* is discharge current (A), Δ*t* is discharge time (s), Δ*V* is potential window (V), and *m* is the mass of active materials (g).

A Ni-MOF/MLG-0.30-based ASC device was fabricated by pairing it with activated carbon (AC), with filter paper acting as the separator in 4 M KOH electrolyte. The mass ratio of the active materials on the positive and negative electrodes was optimized based on Equation (2) to ensure charge balance.

(2) $$\begin{array}{c} m^{+}C^{+}{∆V}^{+}=m^{-}C^{-}{∆V}^{-} \end{array}$$

Here, *m^+^* and *m*^−^ represent the active loadings (g) on the positive and negative electrodes, respectively, *C^+^* and *C*^−^ represent their respective specific capacitances (F·g^−1^), and Δ*V^+^* and Δ*V*^−^ represent the potential ranges (V) for each electrodes. The ASC’s electrochemical performance was evaluated via CV and GCD measurements using a CHI760E workstation in a dual-electrode configuration. The specific capacitance (*Cs*) of the device was also calculated using Equation (1), where *m* represents the combined mass (g) of the active materials on both electrodes.

The energy density (*E*) and power density (*P*) of the ASC were calculated using Equations (3) and (4), respectively:

(3) $$\begin{array}{c} E=\frac{C_{s}\times {\Delta V}^{2}}{2\times 3.6} \end{array}$$

(4) $$\begin{array}{c} P=\frac{E\times 3600}{∆t} \end{array}$$

Here, *E* is energy density (Wh·kg^−1^), *P* is power density (W·kg^−1^), and Δ*t* is discharge time (s).

## 3. Results and Discussion

**Figure 2:**
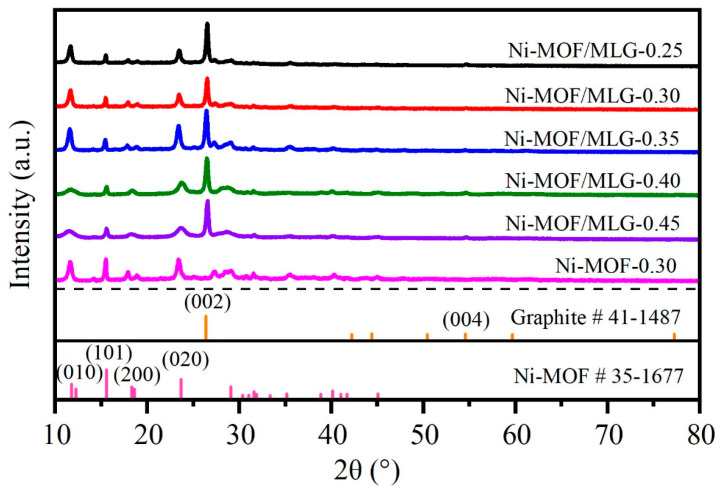
XRD patterns of the samples together with standard patterns.

XRD patterns in Figure 2 characterized the crystalline structure of the prepared samples. Distinct diffraction peaks at 11.8°, 12.3°, 15.6°, 18.3°, 18.6°, 23.7°, 29.1°, 31.6°, 35.2°, 40.1°, and 45.1° correspond to the Ni-MOF phase (JCPDS No. 35-1677). The diffraction peaks observed at 26.4° and 54.5° correspond to the (002) and (004) crystal planes of MLG, respectively (JCPDS No. 41-1487). All Ni-MOF/MLG-x composites exhibit combined diffraction features of both Ni-MOF and MLG, confirming the successful integration of Ni-MOF nanosheets onto the MLG substrate. Notably, Ni-MOF/MLG-0.40 and Ni-MOF/MLG-0.45 display broader peak profiles, and the peak intensities are consistent with the standard Ni-MOF reference, suggesting reduced crystallinity or smaller crystallite sizes. In contrast, Ni-MOF/MLG-0.25, Ni-MOF/MLG-0.30, and Ni-MOF/MLG-0.35 exhibit sharper peaks, with a pronounced intensity enhancement at 11.8° (assigned to the (010) crystal plane). This indicates preferred oriented growth of Ni-MOF. Comparing with Ni-MOF/MLG-0.30, Ni-MOF-0.30 consists exclusively of the Ni-MOF phase, with no characteristic peaks corresponding to MLG being detected.

**Figure 3:**
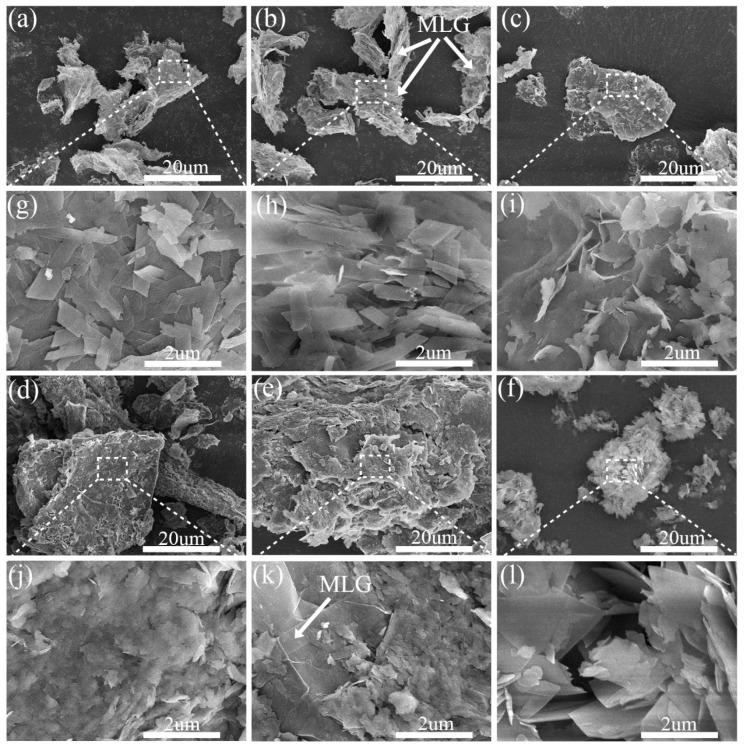
Low- and high-magnification SEM images of the samples: (**a**,**g**) Ni-MOF/MLG-0.25, (**b**,**h**) Ni-MOF/MLG-0.30, (**c**,**i**) Ni-MOF/MLG-0.35, (**d**,**j**) Ni-MOF/MLG-0.40, (**e**,**k**) Ni-MOF/MLG-0.45, (**f**,**l**) Ni-MOF-0.30.

Figure 3 presents SEM images of the Ni-MOF/MLG-x composites, where low-magnification views (Figure 3a–e) reveal the nanosheet structure of MLG substrate, while high-magnification images (Figure 3g–k) demonstrate Ni-MOF anchored onto MLG. Ni-MOF/MLG-0.25, -0.30, and -0.35 exhibit well-defined Ni-MOF nanosheets: parallel-aligned parallelogram nanosheets (0.25), porous stacked parallelogram nanosheets (0.30), and vertically oriented irregular nanosheets (0.35). In contrast, Ni-MOF/MLG-0.40 and -0.45 form nanoparticle films instead of nanosheets, indicating structural degradation at higher precursor concentrations. This morphology evolution arises from nucleation and growth kinetics: lower concentration of NiCl_2_ and PTA favor slow nucleation and growth, enabling ordered nanosheet formation, while higher concentration accelerates nucleation, causing excessive nuclei density and disordered growth. The MLG substrate mitigates Ni-MOF aggregation, as evidenced by the aggregated nanosheets in pure Ni-MOF-0.30 (Figure 3f,l) versus the well-dispersed architecture in Ni-MOF/MLG-0.30, underscoring the role of the MLG in templating controlled growth.

**Figure 4:**
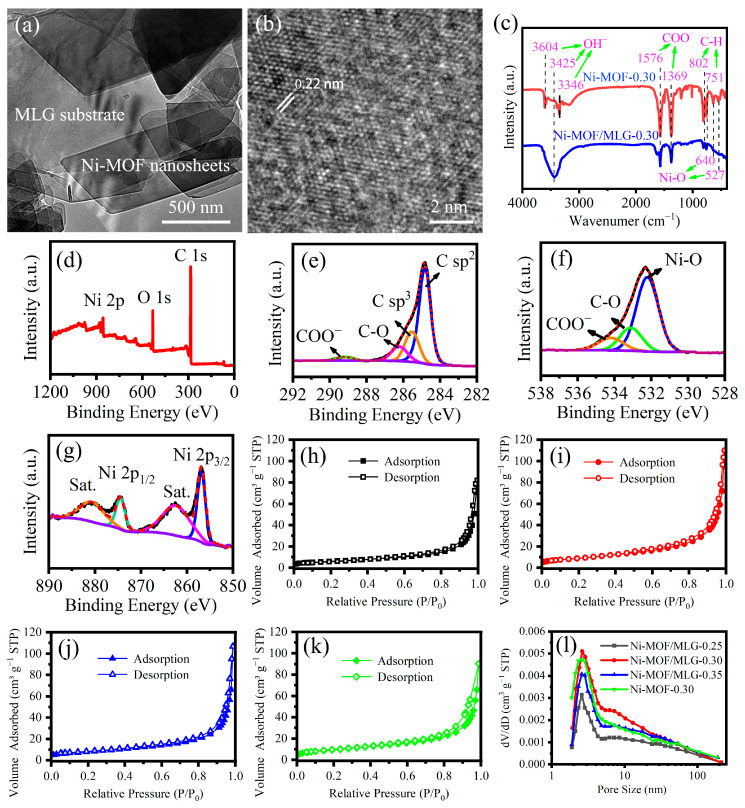
(**a,b**) TEM images of Ni-MOF/MLG-0.30; (**c**) FTIR spectra of Ni-MOF/MLG-0.30 and Ni-MOF-0.30; XPS spectra of Ni-MOF/MLG-0.30: (**d**) Survey, (**e**) C 1s, (**f**) O 1s, and (**g**) Ni 2p; N_2_ adsorption–desorption isotherms: (**h**) Ni-MOF/MLG-0.25, (**i**) Ni-MOF/MLG-0.30, (**j**) Ni-MOF/MLG-0.35, and (**k**) Ni-MOF-0.30; (**l**) Pore size distributions.

Ni-MOF/MLG-0.30 was further characterized via TEM. The TEM images reveal Ni-MOF parallelogram nanosheets distributed on the MLG surface (Figure 4a). HRTEM imaging of the parallelogram nanosheets (Figure 4b) reveals well-defined lattice fringes characteristic of crystalline Ni-MOF, providing the direct evidence for its successful formation on the MLG surface.

Figure 4c presents the FTIR spectra of Ni-MOF/MLG-0.30 and Ni-MOF-0.30. The spectrum of Ni-MOF-0.30 exhibits two sharp peaks at 1377 and 1571 cm^−1^, which are characteristic of the symmetric and asymmetric stretching modes of the COO^−^ groups, respectively. This confirms the bidentate coordination mode of the –COO^−^ groups from PTA to Ni^2+^ [27]. The peaks at 802 and 751 cm^−1^ originate from aromatic C–H bending vibrations. Additionally, the 640 cm^−1^ and 527 cm^−1^ stem from Ni–O vibrational modes. A wide band observed at 3425 cm^−1^ is linked to –OH stretching vibrations from adsorbed H_2_O molecules [27]. These features confirm the structural integrity of Ni-MOF in Ni-MOF-0.30. Notably, all characteristic bands of Ni-MOF are also clearly observed in the Ni-MOF/MLG-0.30 spectrum, providing strong evidence for the successful growth of Ni-MOF on MLG.

Figure 4d–g presents the XPS spectra of Ni-MOF/MLG-0.30. The full-range spectrum reveals that the material consists of elements C, O, and Ni (Figure 4d). In the high-resolution C 1s spectrum (Figure 4e), four distinct peaks at 284.8, 285.5, 286.2, and 289.1 eV can be assigned to the C sp^2^, C sp^3^, C–O, and COO^−^ functional groups [27], respectively. C–O and COO^−^ can be assigned to the PTA molecules in Ni-MOF. C–O and O=C–O bonds are also detected at 533.1 eV and 534.4 eV in the high-resolution O 1s spectrum (Figure 4f), which is consistent with the findings of C 1s spectrum. An additional peak at 532.2 eV in the O 1s spectrum corresponds to Ni–O bonds. In the high-resolution Ni 2p spectrum (Figure 4g), the two spin–orbit splitting peaks at 874.6 eV and 856.7 eV are attributed to the Ni 2p_1/2_ and Ni 2p_3/2_ of Ni^2+^, and the peaks at 880.8 eV and 862.6 eV are their satellite peaks [27]. The XPS analysis provides obvious evidence for the successful synthesis of Ni-MOF in Ni-MOF/MLG-0.30.

To characterize the porous properties of the synthesized materials, nitrogen adsorption–desorption measurements were performed on Ni-MOF/MLG-0.25, -0.30, -0.35, and Ni-MOF-0.30. Figure 4h–k present the N_2_ adsorption–desorption isotherms, which all display characteristic type IV behavior with distinct hysteresis in the high relative pressure region. The specific surface areas are 21.22, 33.58, 28.42, and 32.32 m^2^·g^−1^ for Ni-MOF/MLG-0.25, Ni-MOF/MLG-0.30, Ni-MOF/MLG-0.35, and Ni-MOF-0.30, respectively, with Ni-MOF/MLG-0.30 displaying the highest value. This higher surface area suggests greater availability of active sites for redox reactions. Figure 4l displays the pore size distribution plot of the samples. As observed from Figure 4l, the Ni-MOF/MLG-0.30 sample exhibits a higher distribution intensity in the 5–11 nm pore size range compared to other samples. These mesopores may originate from the interlayer stacking of nanosheets. The presence of such pore sizes is advantageous for electrolyte infiltration during electrochemical processes.

**Figure 5:**
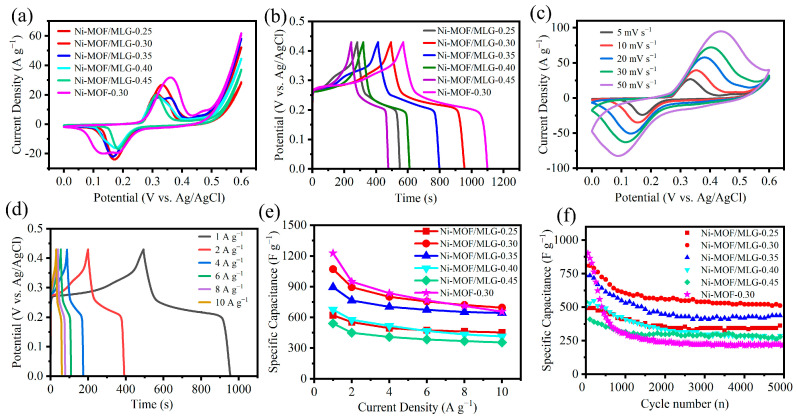
Electrochemical performance of the samples. (**a**) CV curves at 5 mV·s^−1^, (**b**) GCD curves at 1 A·g^−1^, (**c**) CV curves of Ni-MOF/MLG-0.30 at various scan rates, (**d**) GCD curves of Ni-MOF/MLG-0.30 at various current densities, (**e**) Rate performance, (**f**) Cycle performance at 4 A·g^−1^.

Electrochemical characterization reveals well-defined redox peaks in the CV profiles of Ni-MOF/MLG-x composites and Ni-MOF-0.30 (Figure 5a), which can be represented by the following electrochemical equation [27]:

(5) $$\begin{array}{c} {\left[{\mathrm{Ni}}_{3}{\left(\mathrm{OH}\right)}_{2}{\left(C_{8}H_{18}O_{4}\right)}_{2}\cdot H_{2}O\right)}_{4}]\cdot 2H_{2}O+{\mathrm{OH}}^{-}-e^{-}↔[{\mathrm{Ni}}_{3}O(OH){\left(C_{8}H_{18}O_{4}\right)}_{2}\cdot {\left(H_{2}O\right)}_{4}]\cdot 2H_{2}O+H_{2}O] \end{array}$$

Notably, the Ni-MOF/MLG-0.30 composite exhibits the highest current response and largest CV curve area among the Ni-MOF/MLG-x series, indicating its highest specific capacitance. A systematic shift of anodic peaks toward higher potentials is observed from Ni-MOF/MLG-0.25 to Ni-MOF/MLG-0.35, likely due to the increased Ni-MOF nanosheets loading on the MLG surface, which enhances polarization effects. In contrast, Ni-MOF/MLG-0.40 and Ni-MOF/MLG-0.45 display lower anodic peak potentials compared to Ni-MOF/MLG-0.35. This phenomenon may be attributed to the small nanoparticles of Ni-MOF on MLG, which reduce electronic transport distance within the Ni-MOF structure, thereby decreasing polarization. However, despite this advantage, Ni-MOF/MLG-0.40 and Ni-MOF/MLG-0.45 demonstrate lower peak currents than Ni-MOF/MLG-0.30. This observation suggests that the nanosheet architecture of Ni-MOF plays a critical role in achieving higher specific capacitance, likely due to enhanced surface area.

Figure 5b presents the GCD curves, where distinct charge/discharge plateaus confirm pseudocapacitive behavior. The high symmetry of these plateaus suggests excellent Coulombic efficiency. Calculated specific capacitances for Ni-MOF/MLG-0.25, Ni-MOF/MLG-0.30, Ni-MOF/MLG-0.35, Ni-MOF/MLG-0.40, Ni-MOF/MLG-0.45, and Ni-MOF-0.30 are 620.4, 1071.4, 895.1, 676.5, 540.7, and 1227.2 F·g^−1^, respectively. Among the Ni-MOF/MLG-x composites, Ni-MOF/MLG-0.30 demonstrates the highest specific capacitance, likely attributable to its well-crystallized Ni-MOF parallelogram nanosheets with porous architecture. This structure enhances active site availability and facilitates electrolyte infiltration. Ni-MOF-0.30 demonstrates superior specific capacitance compared to Ni-MOF/MLG-0.30, likely stemming from its well-defined nanosheet morphology and the absence of MLG, a non-pseudocapacitive component with small contribution to charge storage. However, this structural advantage is counterbalanced by inferior performance in other electrochemical metrics, such as rate capability or long-term stability, where the MLG-containing composites may excel.

Figure 5c presents the CV curves of Ni-MOF/MLG-0.30 at scan rates ranging from 5 to 50 mV·s^−1^. The CV profiles retain their shape even at elevated scan rates, demonstrating high reversibility of the redox reactions. The anodic peak positively shifts while the cathodic peak negatively shifts, reflecting the polarization effects during charge transfer processes. GCD curves of Ni-MOF/MLG-0.30 measured at different current densities are presented in Figure 5d. The well-maintained symmetry of the GCD curves highlights excellent rate performance. Specific capacitances of Ni-MOF/MLG-0.30, calculated from these GCD curves, are shown in Figure 5e alongside comparative data for other samples. The specific capacitances of Ni-MOF/MLG-0.30 are 1071.4, 895.3, 800.9, 754.9, 721.9, and 698.3 F·g^−1^ at current densities of 1, 2, 4, 6, 8, and 10 A·g^−1^, respectively. Capacitance retention rates at 10 A·g^−1^ are 73.1%, 64.8%, 71.9%, 61.2%, 65.8%, and 53.5% for Ni-MOF/MLG-0.25, Ni-MOF/MLG-0.30, Ni-MOF/MLG-0.35, Ni-MOF/MLG-0.40, Ni-MOF/MLG-0.45, and Ni-MOF-0.30, respectively. The Ni-MOF/MLG-0.30 sample exhibits slightly lower rate performance compared to Ni-MOF/MLG-0.25 and Ni-MOF/MLG-0.35. This could be attributed to the stacked configuration of nanosheets, which likely introduces extended electron transport pathways and increased resistance during charge/discharge processes. Notably, Ni-MOF/MLG-0.30 exhibits superior rate performance compared to Ni-MOF-0.30, indicating that the MLG substrate enhances rate capability due to its high electrical conductivity.

The cycling performance of the samples at 4 A·g^−1^ is shown in Figure 5f. After 5000 cycles, Ni-MOF/MLG-0.25, Ni-MOF/MLG-0.30, Ni-MOF/MLG-0.35, Ni-MOF/MLG-0.40, Ni-MOF/MLG-0.45, and Ni-MOF-0.30 retain 68.5%, 63.2%, 60.7%, 53.7%, 65.9%, and 23.7% of their initial capacitances, respectively. The capacity fade can be attributed to partial structural degradation during prolonged charge–discharge cycling. Ni-MOF/MLG-0.30 demonstrates superior capacitance retention compared to its MLG-free counterpart (Ni-MOF-0.30), implying that MLG alleviates structural damage to Ni-MOF during redox reactions. This enhancement arises from the dual functionality of MLG as both a flexible conductive scaffold and a mechanical buffer, which synergistically stabilizes the active material while preserving efficient charge transport pathways. Since Ni-MOF and MLG are bonded via molecular interactions, the contact area has an influence on the cycling stability of the composite. In Ni-MOF/MLG-0.25, the Ni-MOF nanosheets exhibit parallel alignment to the MLG surface, while Ni-MOF/MLG-0.45 features nanoparticle films deposited on MLG. Both configurations maximize the Ni-MOF/MLG interfacial contact area, thereby enhancing inter-component interactions and consequently improving cycling stability compared to Ni-MOF/MLG-0.30. However, this structural advantage comes at the expense of specific capacity, which is significantly lower than that of Ni-MOF/MLG-0.30. Thus, Ni-MOF/MLG-0.30’s porous stacked nanosheet architecture achieves an optimal compromise between these competing factors, ultimately delivering the best overall electrochemical performance. The electrochemical performance of our prepared Ni-MOF/MLG-0.30 composite is compared with reported Ni-MOF-based materials in Table 1. As shown in the table, Ni-MOF/MLG-0.30 exhibits high specific capacitance and rate capability. However, its cycling stability requires further improvement, which will be addressed in our future research.

Charge storage arises from two distinct mechanisms: diffusion-controlled processes and capacitive (surface-controlled) contributions. These contributions can be quantified using Equation (6) [30]. Its derived Equation (7) indicates the kinetic parameter *b* can be determined through linear regression analysis of log(*i*) versus log(*v*) plots. The *b* value provides insight into the dominant mechanism: *b* = 0.5 indicates a dominant diffusion-controlled Faradaic process, while *b* = 1 corresponds to a purely capacitive surface-controlled behavior. Intermediate *b* values (0.5 < *b* < 1) indicate a hybrid process involving both diffusion- and surface-controlled contributions.

(6) $$\begin{array}{c} i=av^{b} \end{array}$$

(7) $$\begin{array}{c} log\left(i\right)=log\left(a\right)+blog\left(v\right) \end{array}$$

**Figure 6:**
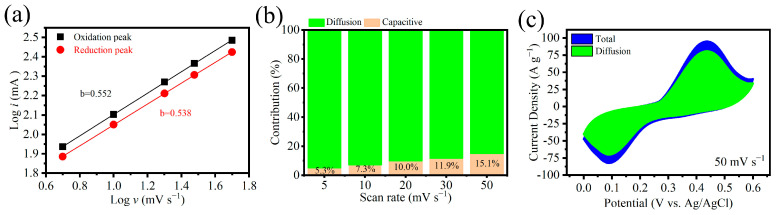
(**a**) log (i) vs. log (*v*) for calculation of the b value of the Ni-MOF/MLG-0.30 electrode. (**b**) Capacitive- and diffusion-controlled contributions of Ni-MOF/MLG-0.30 at various scan rates. (**c**) Diffusive contributions plotted in CV curves at 50 mV·s^−1^.

Figure 6a shows the plots of log(*i*) vs. log(*v*) from the anodic and cathodic peaks of Ni-MOF/MLG-0.30. *b* values calculated from the plots are 0.552 and 0.538, respectively, indicating diffusion-dominated processes.

The total current can be expressed as the sum of a capacitive-controlled component (*k*_1_*v*) and a diffusion-controlled component (*k*_2_*v*^1/2^), as formulated in Equation (8). Equation (9) is derived by rearranging Equation (8), revealing a linear relationship between *i*/*v*^1/2^ and *v*^1/2^ with a slope of *k*_1_. The capacitive contribution can then be quantified using the term *k*_1_*v*/*i*.

(8) $$\begin{array}{c} i=k_{1}v+k_{2}v^{1/2} \end{array}$$

(9) $$\begin{array}{c} i/v^{1/2}=k_{1}v^{1/2}+k_{2} \end{array}$$

Figure 6b illustrates the relative contributions of capacitive- and diffusion-controlled processes in Ni-MOF/MLG-0.30 across varying scan rates. The capacitive contributions are 5.3%, 7.3%, 10.0%, 11.9%, and 15.1% at the scan rates of 5, 10, 20, 30, and 50 mV·s^−1^. Capacitive behavior stems from surface-mediated charge storage, while diffusion-controlled contributions arise from redox reactions within the bulk electrode material. At higher scan rates, the limited time available for redox reactions to fully proceed results in a decrease in the diffusion-controlled contribution. Figure 6c further highlights the dominance of the diffusion-controlled contribution at 50 mV·s^−1^, where the shaded green region (diffusion-controlled contribution) constitutes 84.9% of the total CV curve area (blue region).

**Figure 7:**
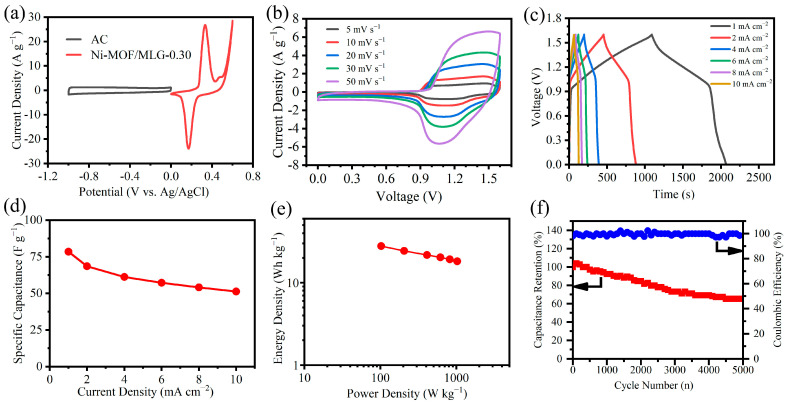
(**a**) CV curves of AC and Ni-MOF/MLG-0.30 electrodes at 5 mV·s^−1^, (**b**) CV curves of the ASC device at different scan rates, (**c**) GCD curves at the different current densities, (**d**) Rate performance, (**e**) Ragone plot, (**f**) Cycle performance at 4 mA·cm^−2^.

Practical electrochemical performance was evaluated by assembling an asymmetric supercapacitor (ASC) using Ni-MOF/MLG-0.30. As illustrated in Figure 7a, the CV curves of Ni-MOF/MLG-0.30 and activated carbon (AC) display characteristic pseudocapacitive behavior (redox peaks) and electric double-layer capacitance (rectangular shape), respectively. Thus, their integration enables the ASC device to be worked in an extended voltage range of 0–1.6 V (Figure 7b). The ASC device was characterized through CV measurements at multiple scan rates. The CV curves maintain their characteristic shape without significant deformation at elevated scan rates up to 50 mV·s^−1^ (Figure 7b), demonstrating outstanding rate performance. Figure 7c presents the GCD profiles of the ASC measured under different current densities. It exhibits symmetrical triangular charge–discharge behavior, reflecting high Coulombic efficiency and robust rate performance. The specific capacitances, calculated from the GCD data and summarized in Figure 7d, demonstrate current-density-dependent values of 78.5, 68.6, 61.2, 57.2, 54.1, and 51.3 F·g^−1^ at 1, 2, 4, 6, 8, and 10 mA·cm^−2^, respectively. The Ragone plot (Figure 7e) demonstrates that the ASC delivers an energy density of 27.9 Wh·kg^−1^ at 102.5 W·kg^−1^ and retains 18.2 Wh·kg^−1^ at 1024.6 W·kg^−1^, highlighting its suitability for high-power applications. Furthermore, after 5000 cycles at 4 mA·cm^−2^, the ASC maintains 63.5% of its initial capacitance while maintaining a coulombic efficiency above ~98% throughout the entire process (Figure 7f), demonstrating a possibility for real-world energy storage systems.

## 4. Conclusions

In summary, nickel-based metal–organic frameworks (Ni-MOF) are deposited onto mechanically exfoliated multilayer graphene (MLG) via van der Waals-assisted chemical bath deposition to fabricate high-performance supercapacitor electrodes. The resulting Ni-MOF/MLG-0.30 composite features uniformly distributed, loosely packed Ni-MOF parallelogram nanosheets on the MLG substrate, a morphology that enhances electrochemical performance by facilitating ion/electron transport. The conductive MLG framework improves charge transfer efficiency within the composite, while the porous and well-dispersed Ni-MOF nanostructure maximizes accessible active sites and electrolyte accessibility. The Ni-MOF/MLG-0.30 electrode demonstrates a specific capacitance of 1071.4 F·g^−1^ at 1 A·g^−1^ and retains 63.2% of its initial capacitance after 5000 charge/discharge cycles at 4 A·g^−1^. This work presents a scalable and efficient strategy for designing advanced supercapacitor electrodes through the strategic coupling of MOFs with mechanically exfoliated graphene, highlighting the synergy between tailored MOF nanostructures and conductive supports.

## Figures and Tables

**Table 1:** List of the electrochemical performance of Ni-MOF-based materials.

Material	Electrolyte	Capacitance (F·g^−1^) @Current Density (A·g^−1^)	Rate Capability @Current Density (A·g^−1^)	Cycling Stability/Cycles @Current Density (A·g^−1^)	Ref.
Ni-MOF thin films on steel	1.0 M KOH	850.4@ 1 mA·cm^−2^	88.2% @5 mA·cm^−2^	70.2%/3000 @1 mA·cm^−2^	[11]
Ni-MOF/rGO	6.0 M KOH	954.0@1	80.3%@10	80.2%/4000@5	[19]
Ni-MOF/MWCNT	6.0 M KOH	900.0@0.5	29.5%@8	82.0%/5000@2	[25]
GM LEG@Ni-MOF	3.0 M KOH	987.6@0.5	58.7%@10	85.6%/3000@5	[27]
Ni-MOF/GO	1.0 M KOH	630.0@1	69.0%@10	56.5%/10000@5	[28]
Ni-MOF	1.0 M KOH	941.0@1	53.5%@10	78.5%/3000@5	[29]
Ni-MOF/MLG-0.30	4.0 M KOH	1071.4@1	64.9%@10	62.3%/5000@4	this work

## Data Availability

The original contributions presented in this study are included in the article. Further inquiries can be directed to the corresponding authors.
